# Oncogenic Signalling of Growth Factor Receptors in Cancer: Mechanisms and Therapeutic Opportunities

**DOI:** 10.3390/ijms23137376

**Published:** 2022-07-02

**Authors:** Anica Dricu

**Affiliations:** Department of Biochemistry, Faculty of Medicine, University of Medicine and Pharmacy, 200349 Craiova, Romania; anica.dricu@live.co.uk

Cancer is a common name for several distinct diseases caused by uncontrolled cell growth and proliferation. More than 200 types of cancer are described in the literature, each of them with its own identity given by specific gene, protein or hormone signatures. However, concerted and redundant dysregulations of mitogenic pathways arising from growth factor receptors (GFRs) are common events in all cancer types [1,2].

These sophisticated membrane-spanning proteins harmonize the information flow from several sources, controlling the mitogenic network in the normal cell. The complexity of GFRs function is supported by their multiple regulatory mechanisms, including feedback loops, multidirectional cross-communication and redundancy in downstream signalling. Recent large-scale studies identified alterations in genes and proteins of several GFRs such as epidermal growth factor receptor (EGFR), platelet-derived growth factor receptor α/β (PDGFR α/β), vascular endothelial growth factor receptors (VEGFRs), IGF-1R, fibroblast growth factor receptor (FGFR), etc. [3].

The majority of malignant diseases are related to aberrant intra- and intercellular communication, associated with subverted GFRs pathways. At the molecular level, the overactivation of GFRs induces a mitogenic response and maintains cancer cell growth. Four main mechanisms are known to generate aberrant activation of GFRs in malignant diseases: autocrine/paracrine activation, genomic amplification, chromosomal rearrangements and gain-of-function mutations [4,5].

GFs mediate their mitogenic function by binding to and activating GFRs with intrinsic tyrosine kinase (TKs) activity. Cancer cells produce GFs or reprogram and force other cells to produce GFs according to their own needs, becoming independent of endocrine signalling and finally leading to constitutive receptors activation in tumours [6,7,8].

GFRs gene amplification, also known as genomic DNA copy number amplification, has been found in a wide variety of tumours, causing receptor protein upregulation and overactivation, inducing oncogenic behaviour and resistance to therapy [9,10].

Chromosome rearrangements mechanism is a usual condition of malignant cells, in which a fragment of chromosomes is deleted or inverted, giving rise to fusion proteins that are responsible for the formation of several types of malignancies. The BCR-ABL fusion oncoprotein, which fuses the ABL1 tyrosine kinase gene on chromosome 9 to the BCR gene on chromosome 22, was the first tyrosine kinase fusion identified [11]. Chromosome rearrangements leading to fusion proteins are also found in many solid cancers, such as breast cancer, brain tumours, lung cancer, colorectal cancer, etc. [12,13,14,15].

Gain-of-function mutations can exercise mitogenic functions by stimulation of growth factors or by inducing constitutive activation of GFRs, driving uncontrolled cell proliferation and tumour progression [16].

Once activated, GFRs trigger a wave of intracellular signalling events, mediated by two major pathways: mitogen-activated protein kinase (MAPK) and phosphoinositide 3-kinases (PI3K) cascades [17].

Many intracellular proteins involved in rat sarcoma virus (RAS)/MAPK or PI3K/AKT pathways can also function as oncogenes. Mutations affecting key proteins in RAS/MAPK or PI3K/AKT pathways are known to be crucial in maintaining the malignancy of different types of cancers [18,19,20].

Many effector proteins in GFRs signal transduction, such as PI3K, extracellular signal-regulated kinase 1/2 (ERK1/2) or MAPK can act as junction for multiple signalling pathways [21]. It is also well demonstrated that mutations in mammalian target of rapamycin (mTOR), RAS or rapidly accelerated fibrosarcoma (RAF) are very common in malignant diseases [22].

Crosstalk and collaboration between GFRs and other protein families are constantly being discovered, making the receptor signalling system far more complex. For example, G protein-coupled receptors (GPCRs) can engage GFRs to mediate cell proliferation, differentiation, and vice versa, several GFs use GPCRs proteins to exert their mitogenic signal signalling [23].

Moreover, the evasion of apoptotic signals and the requirement of angiogenesis were also found to be of fundamental importance for tumour progression and metastasis. In this context, high expression of GFRs aids blood vessel formation, cell migration and the inhibition of apoptosis [24,25].

All this information has guided the development of compounds, designed to target one or more of these pathways in cancer cells. A vast variety of GFR signalling inhibitors have been developed, many of which have been approved by the Food and Drug Administration (FDA). While some FDA-approved inhibitors are selective for individual GFRs (e.g., Alectinib, Afatinib, Dacomitinib, Erlotinib, Gefitinib, Lapatinib, etc.), others demonstrate efficiency by inhibiting several receptors (e.g., Dasatinib, Lestaurtinib, Imatinib, Ponatinib, Vandetanib, etc.). However, the development of novel therapeutic strategies for cancer treatment is tightly restricted by the similarities between the normal and malignant cells. GFR-directed therapy that would theoretically selectively kill malignant cells and reduce the toxicity associated with nonselective conventional chemotherapy may be a promising treatment for cancer. Based on this rationale, different strategies have been developed to inhibit the oncogenic effects of GFRs (e.g., small-molecule inhibitors, monoclonal antibodies, siRNA, antisense oligodeoxynucleotides, triple helix, dominant-negative mutants, etc.).

This Special Issue will cover the latest preclinical and clinical progress made in the areas associated with GFRs’ oncogenic signalling.
